# Seebeck Coefficient of Thermocouples from Nickel-Coated Carbon Fibers: Theory and Experiment

**DOI:** 10.3390/ma11060922

**Published:** 2018-05-30

**Authors:** Hardianto Hardianto, Gilbert De Mey, Izabela Ciesielska-Wrόbel, Carla Hertleer, Lieva Van Langenhove

**Affiliations:** 1 Department of Materials, Textiles and Chemical Engineering, Ghent University, Technologiepark 907, 9052 Zwijnaarde, Belgium; budysiowka@gmail.com (I.C.-W.); carla.hertleer@gmail.com (C.H.); Lieva.VanLangenhove@UGent.be (L.V.L.); 2 Department of Textile Chemistry, Politeknik STTT Bandung, Jalan Jakarta 31, Bandung 40272, Indonesia; 3 Department of Electronics and Information Systems, Ghent University, Technologiepark 15, 9052 Zwijnaarde, Belgium; Gilbert.DeMey@UGent.be

**Keywords:** thermocouple, Seebeck coefficient, conductive yarn, nickel-coated carbon fiber

## Abstract

Thermocouples made of etched and non-etched nickel-coated carbon yarn (NiCCY) were investigated. Theoretic Seebeck coefficients were compared to experimental results from measurements of generated electric voltage by these thermocouples. The etching process for making thermocouples was performed by immersion of NiCCY in the solution containing a mixture of hydrochloric acid (HCl) (37% of concentration), and hydrogen peroxide (H_2_O_2_) in three different concentrations—3%, 6%, and 10%. Thirty minutes of etching to remove Ni from NiCCY was followed by washing and drying. Next, the ability to generate electrical voltage by the thermocouples (being a junction of the etched and the non-etched NiCCY) was measured in different ranges of temperatures, both a cold junction (291.15–293.15 K) and a hot junction (293.15–325.15 K). A formula predicting the Seebeck coefficient of this thermocouple was elaborated, taking into consideration resistance values of the tested samples. It was proven that there is a good agreement between the theoretical and experimental data, especially for the yarns etched with 6% and 10% peroxide (both were mixed with HCl). The electrical resistance of non-fully etched nickel remaining on the carbon fiber surface ($R_{1}$) can have a significant effect on the thermocouples’ characteristics.

## 1. Introduction

Smart textiles development has entered a new stage where all the electronic components previously incorporated with textile elements are gradually replaced by electronic-like textiles components, e.g., textile capacitors [1], highly flexible textile antennas [2]. This means that electronic components are usually replaced by pliable and limp films, yarns, fibers, conductive coatings, printed electronics, etc., to make these new smart textiles stretchable, washable, durable and lasting. These last three features have been playing an important role in smart textiles release to the market and the fact that these three conditions have not been fulfilled is exactly why one may not observe all the interesting innovative smart textiles solutions on the market.

This research work is aimed at building a reliable textile thermocouple on a linear textile product (yarn) that could be incorporated as weft, with a thin fabric being a stratum. This stratum that separates two zones provides information on measurements of temperature differences between these two zones. In order to create a textile thermocouple, a junction of two conductive yarns has to be created. In fact, any thermocouple can be created by making a proper circuit with two different conductors that allow the generation of an electric voltage. This electric voltage is known as the Seebeck effect (S). Its efficiency depends on a ratio of the generated electric voltage and a temperature difference between environments where each of the conductors’ join [3].

There are several known solutions attempting textile application of the thermocouple principal presented by other authors, e.g., using constantan with steel to create a thermocouple [4], and constantan with copper for the same purpose [5]. Thermocouples were integrated into textile substrates by researchers as a temperature sensor [6] and heat flux sensor [7,8,9]. However, there is a disadvantage to the application of these sorts of materials because they are brittle and consequently break easily. Additionally, metal wires integrated into textiles as thermocouples affect the flexibility of these textiles. Therefore, finding textile-based conductive yarns for fabricating a thermocouple and a thermopile for wearable textiles is of great interest.

Recently, huge improvements of the Seebeck coefficient have been found by using other forms of carbon such as carbon nanotube and graphene [10,11,12]. However, these promising materials are not available in textile yarns which can be inserted into wearable textiles. The toxicity of carbon nanotubes is also a problem. Therefore, nickel-coated carbon yarn (NiCCY) can be a good candidate material for fabricating a textile-based thermoelectric generator.

From our preliminary experiment, a thermocouple from two different conductive textile yarns i.e., carbon fiber and NiCCY demonstrated a Seebeck coefficient of about 18 µV/K. According to this result, there is a possibility to create a thermopile using NiCCY. Carbon fiber coated with nickel is also available on the market. This led us to an idea to create a textile-based thermopile from a single NiCCY, provided that the nickel can be removed selectively to form a series of C-Ni junctions along the NiCCY forming a thermopile to achieve higher voltage output since the voltage generated by a thermopile is proportional to the number of thermocouple junctions. The previous report showed that mixture of peroxide and hydrochloric acid can significantly remove the nickel from nickel-coated carbon fiber [13].

Textile-based conductive yarns are sought for their integration into the textile structures to act as thermocouples or thermopiles, to create more flexible textile-based temperature- or heat flux sensors. There is a great potential for making thermocouples based on existing carbon yarns, especially with Tenax^®^-J HTS40 A23 12K 1420tex provided by Toho Tenax Europe GmbH, Germany, which has a Ni coating [13]. In this nickel-coated carbon yarn (NiCCY), both C and Ni are electric conductors. Tenax^®^ refers to a group of high-performance carbon fibers. The high tenacity (HT) fibers provide excellent mechanical laminate properties. This fiber is manufactured from a polyacrylonitrile (PAN) precursor and is surface-treated in order to promote adhesion to organic matrix polymers [14].

In this paper, we are going to study the effect of the electrical resistance of the etched NiCCY on the Seebeck coefficient of the thermocouple made of the etched NiCCY and non-etched because it is easier to measure the electrical resistance than to measure the thickness of the nickel layer on NiCCY which is less than 0.25 µm. A formula for predicting the Seebeck coefficient of this thermocouple is going to be elaborated by taking into consideration the resistance values of the yarns. This theoretical formula will be compared to the experimental results.

**Figure 1:**
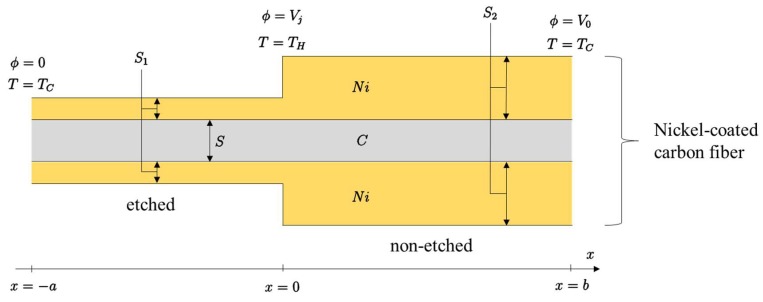
Theoretical diagram of etched and non-etched segment of one filament of nickel-coated carbon fiber as a thermocouple where *C* is carbon fiber; *Ni* is nickel coating on the surface of *C* fiber; *S*, *S*_1_, and *S*_2_ are the cross-section areas of *C*, *Ni* on etched segment and *Ni* on non-etched segment, respectively; *T* is temperature; *T_C_* and *T_H_* are cold and hot temperatures, respectively; *ϕ* is electric potential; *V_j_* is a voltage at junction (*x* = 0); *V*_0_ is a voltage at a single end of nickel-coated carbon fiber (*x* = b); *x* is axis along the thermocouple.

The electric conductivity σ  of the nickel is much higher compared to carbon ($\sigma_{Ni}=14.3\times 10^{6}\;S/m$ vs. $\sigma_{C}=5.9\times 10^{6}\;S/m$) [15]. In order to make a thermocouple, we have to use two different materials. In this work, this will be performed by etching the Ni layers from the part of the NiCCY, as schematically represented in Figure 1. Both ends (x=−a and x=b) are at the “cold” temperature, $T_{C}$, which is normally the ambient room temperature. The junction (*x* = 0) between the etched and the non-etched part is at a higher temperature, $T_{H}$. At both cold ends, metallic contacts are provided to measure the generated voltage, $V_{0}$.

Figure 1 demonstrates an idealised situation in which a single fiber is partially etched, thus having two different amounts of Ni on its surface. The idealised and simplified situation where two different thicknesses of Ni on carbon fiber (CF) are presented, makes the mathematical modeling of a theoretical approach transparent.

Theoretically, the most efficient thermocouple is created if a significant part or the whole of the layer of Ni is removed from the surface of CF during the etching process. During the etching process, it was possible to remove an unknown amount of Ni so that when the periodically etched yarn and the non-etched yarn were connected to the voltage meter, the junction allowed for the generation of an output voltage of about 18 µV/K. The etching process and the subsequent measurements were performed on the whole yarn (all the fibers in the yarn were treated at the same time). It would be difficult to treat a single fiber individually. Due to the higher electrical conductivity of Ni, the right part of the fiber section might seem to be made from pure nickel and the presence of carbon may be overlooked. Occasionally, it might happen that not all the nickel has been removed from the part that was intentionally subject to etching so that the performance of the thermocouple might be reduced.

## 2. Materials and Methods

### 2.1. Material

**Figure 2:**
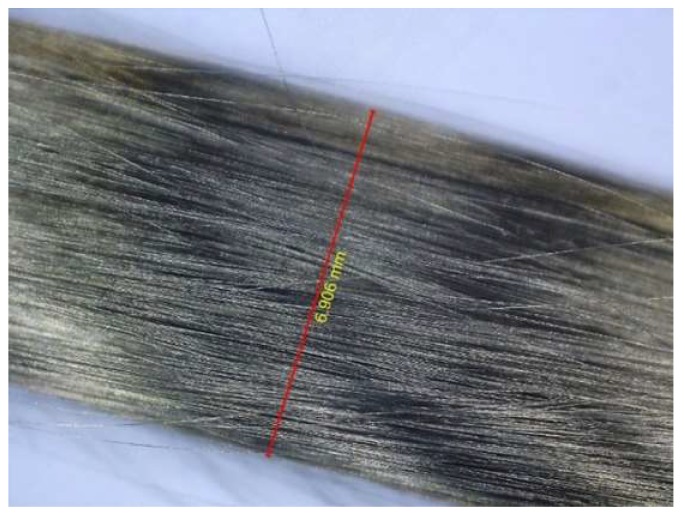
An image of Tenax^®^-J HTS40 1420tex NiCCY provided by Toho Tenax Europe GmbH, Wuppertal, Germany. The image was taken with OneBird Smart 5M 300X USB Digital Microscope Camera Video with MicroCapture. The diameter of 6.906 mm was measured without any pretension [14].

Materials used in this experiment were similar to previous work [13]. The NiCCY used in this experiment is Tenax^®^-J HTS40 A23 12K 1420tex MC from Toho Tenax Europe GmbH, Wuppertal, Germany. Figure 2 shows the yarn and Table 1 presents its parameters.

### 2.2. Etching Process

The process of removing Ni in this experiment is called an etching process. Combination of HCl and H_2_O_2_ solution was utilized to remove Ni from NiCCY and is then called etching solution. The H_2_O_2_ concentration varied from 3%, to 6% up, and to 10%, while HCl was kept constant at 37%. The ratio between H_2_O_2_ and HCl was 1:1. The samples were immersed in the etching solutions for 30 min. Next, the sample was washed with water to remove the remaining chemicals from the yarn. The excess of water remaining on the sample was absorbed by blotting paper several times and all the samples were air dried at room temperature for a minimum of 24 h prior to testing [13].

### 2.3. Seebeck Coefficient Measurement

**Figure 3:**
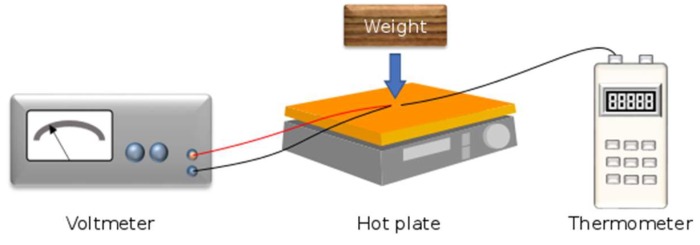
Illustration of the voltage measurement set up. The junction was placed on the hot plate that had been covered with a piece of paper and a wood weight was placed on the junction and thermometer probe.

The electric voltage of the thermocouple samples was measured using a nanovoltmeter Amplificateur NV 724 from Setaram, Lyon, France. A Fluke 52 digital thermometer (Fluke, Everett, Washington, DC, USA) was used to measure the temperature during voltage measurement. The higher temperature range for the hot junction was controlled by an electric hot plate, while the lower temperature range for the cold junction was dependent on the external conditions of the laboratory, which were controlled and stable. Figure 3 shows the voltage measurement set up of etched and non-etched NiCCY.

The efficiency of the thermocouple was measured through different ranges of temperatures for a cold junction (291.15–293.15 K) and for a hot junction (293.15–325.15 K). These temperature ranges were the only ones enabling the performance of stable and repeatable measurements.

### 2.4. Theoretical Analysis

The following theoretical calculations are formulated according to the schematic diagram of NiCCY that has been shown in Figure 1 earlier. Generally, the temperature varies along the thermocouple wire: *T*(*x*). We know that *T*(−*a*) = *T*(*b*) = *T_C_* and *T*(0) = *T_H_*. Similarly, the electric potential ϕ (x) depends on *x*. The generated voltage can be calculated as:

(1) $$V_{0}=\phi \;\left(b\right)-\phi \;\left(-a\right)$$

Inside each conductor, the current density J (expressed in $A/m^{2})$ is given from literature [16]:

(2) $$J=-\sigma \left(\frac{d\phi }{dx}+\varepsilon \frac{dT}{dx}\right)$$

where *σ* is the electric conductivity and *ε* the Seebeck coefficient. From the literature, the numerical values are *ε_Ni_* = −14.8 µV/K and *ε_C_* = +3.0 µV/K [17]. Hence, the value 14.8 + 3 = 17.8 µV/K is the highest value one can obtain with a carbon-nickel thermocouple. The electric current *I*_1_ flowing through the left part is then:

(3) $$I_{1}=-\sigma_{C}\left(\frac{d\phi }{dx}+\varepsilon_{C}\frac{dT}{dx}\right)S-\sigma_{Ni}\left(\frac{d\phi }{dx}+\varepsilon_{Ni}\frac{dT}{dx}\right)S_{1}$$

Similarly, for the right part, we have to replace $S_{1}$ by $S_{2}$ to get $I_{2}$:

(4) $$I_{2}=-\sigma_{C}\left(\frac{d\phi }{dx}+\varepsilon_{C}\frac{dT}{dx}\right)S-\sigma_{Ni}\left(\frac{d\phi }{dx}+\varepsilon_{Ni}\frac{dT}{dx}\right)S_{2}$$

The thermocouple voltage is measured with a meter having a high input impedance. Hence, almost no current can flow or:

(5) $$I_{1}=I_{2}=0$$

Integrating (3) with respect to x gives then:

(6) $$\sigma_{C}\left(\phi +\varepsilon_{C}T\right)S+\sigma_{Ni}\left(\phi +\varepsilon_{Ni}T\right)S_{1}=A$$

where *A* is an integration constant. Applying (6) in the point x=−a and x=0 gives, after subtraction:

(7) $$\sigma_{C}\left(V_{j}+\varepsilon_{C}T_{H}\right)S+\sigma_{Ni}\left(V_{j}+\varepsilon_{Ni}T_{H}\right)S_{1}-\sigma_{C}\varepsilon_{C}T_{C}S-\sigma_{Ni}\varepsilon_{Ni}T_{C}S_{1}=0$$

or:

(8) $$\left(\sigma_{C}S+\sigma_{Ni}S_{1}\right)V_{j}+\left(\sigma_{C}\varepsilon_{C}S+\sigma_{Ni}\varepsilon_{Ni}S_{1}\right)\left(T_{H}-T_{C}\right)=0$$

A similar calculation for the right part (0<x<b) gives us:

(9) $$\left(\sigma_{C}S+\sigma_{Ni}S_{2}\right)\left(V_{j}-V_{0}\right)+\left(\sigma_{C}\varepsilon_{C}S+\sigma_{Ni}\varepsilon_{Ni}S_{2}\right)\left(T_{H}-T_{C}\right)=0$$

Elimination of the junction potential $V_{j}$ from (8) and (9) gives us finally:

(10) $$V_{0}=\left(T_{H}-T_{C}\right)\left[\frac{\sigma_{C}\varepsilon_{C}S+\sigma_{Ni}\varepsilon_{Ni}S_{2}}{\sigma_{C}S+\sigma_{Ni}S_{2}}-\frac{\sigma_{C}\varepsilon_{C}S+\sigma_{Ni}\varepsilon_{Ni}S_{1}}{\sigma_{C}S+\sigma_{Ni}S_{1}}\right]$$

Rewriting (10) gives:

(11) $$V_{0}=\left(T_{H}-T_{C}\right)\frac{\sigma_{C}\sigma_{Ni}S\left(S_{2}-S_{1}\right)}{\left(\sigma_{C}S+\sigma_{Ni}S_{2}\right)\left(\sigma_{C}S+\sigma_{Ni}S_{1}\right)}\left(\varepsilon_{Ni}-\varepsilon_{C}\right)$$

As expected, we obtain an output voltage $V_{0}$ proportional to the temperature difference $\;T_{H}-T_{C}$. Obviously, the result of Equation (11) is also proportional to the difference of the two Seebeck coefficients,$\;\varepsilon_{Ni}-\varepsilon_{C}$.

If $S_{1}=0$, or Ni has been completely removed from the left part, and $\sigma_{Ni}S_{2}\gg \sigma_{C}S$, the relation (11) can be simplified to:

(12) $$V_{0}=\left(T_{H}-T_{C}\right)\left(\varepsilon_{Ni}-\varepsilon_{C}\right)$$

which is the formula we are most familiar with. Note that the term (12) is also the highest value one can obtain with a C-Ni thermocouple.

## 3. Results and Discussion

### 3.1. Microscopic Observation after Etching Process

**Figure 4:**
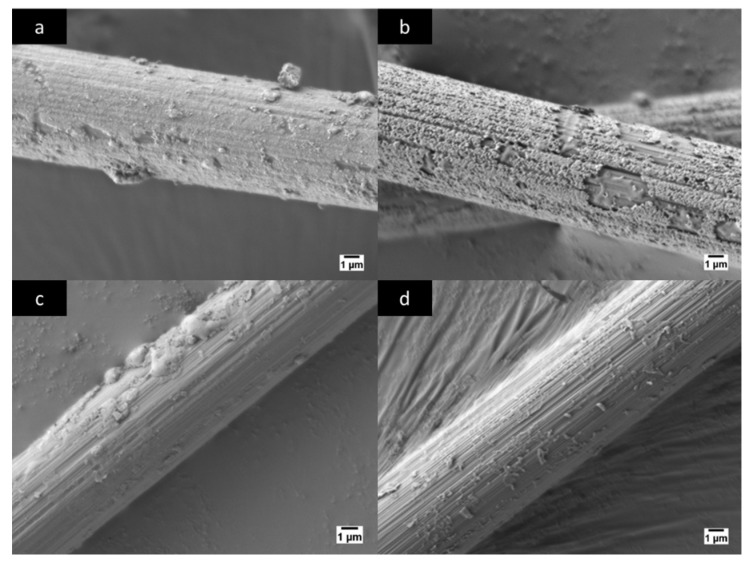
Images from scanning electron microscope (Jeol JSM-7600F) taken with 5000x magnification: (**a**) Untreated nickel-coated carbon fiber; (**b**) nickel-coated carbon fiber after treatment of 3% H_2_O_2_ + 37% HCl; (**c**) nickel-coated carbon fiber after treatment of 6 % H_2_O_2_ + 37% HCl; (**d**) nickel-coated carbon fiber after treatment of 10% H_2_O_2_ + 37% HCl.

After the chemical treatment of the samples of NiCCY, one observed the effect of the etching process excreted on the treated samples through scanning electron microscope (SEM) image. An untreated sample of NiCCY in Figure 4a was compared to the treated samples in Figure 4b–d where the treatment was 37% HCl and 3%, 6% and 10% of H_2_O_2_, respectively.

Based on the application of different chemical concentration treatments of H_2_O_2_ to the NiCCY and the microstructure (topography) of untreated and treated NiCCY observed via SEM as shown in Figure 4a–d, one may draw a conclusion concerning the potential impact of the treatment. Namely, the higher the concentration of H_2_O_2_, the more intense the etching process was, with less Ni remaining on the surface of C. An obvious difference may be noticed between untreated NiCCY (Figure 4a) and the treated one (Figure 4b), where the H_2_O_2_ concentration was only 3%. In the case of this treatment, the surface of Ni was visibly incised, generating a porous-like layer on the surface of CF. Additionally, some zones of notches on the surface of NiCCY are also visible. This image confirms that in the initial phase, the etching is not homogeneous along the entire yarns, which will also influence the electrical resistance and thermocouple characteristics.

In Figure 4c, one may observe a further etch on the Ni layer due to an increased concentration of H_2_O_2_ from 3% up to 6%_._ Although the concentration of H_2_O_2_ is higher in the case of the treatment presented in Figure 4c, there are still some larger islands of Ni clearly visible. Nevertheless, a large zone of clean and grooved CF is also visible. In the case of the highest utilized concentration of H_2_O_2_ in this experiment, only some small dust, sparsely distributed, are present on the surface of CF (Figure 4d). Therefore, this confirms that the sample treated in 10% H_2_O_2_ + 37% HCl can be considered as a fully etched yarn.

### 3.2. Comparison between Experiment and Theory

In order to check the theoretical analysis, it is more convenient to use resistance values of the yarns to be inserted in (11). The reason is obvious; resistance can be easily measured whereas a cross section like S, $S_{1}$ or $S_{2}$ are hard to obtain.

The following resistances (expressed in Ω/m) are defined:

(13) $$R_{C}=\frac{1}{\sigma_{C}S}$$

(14) $$R_{1}=\frac{1}{\sigma_{Ni}S_{1}}$$

(15) $$R_{2}=\frac{1}{\sigma_{Ni}S_{2}}$$

Equation (11) is then rewritten as:

(16) $$V_{0}=\left(T_{H}-T_{C}\right)\frac{R_{C}\left(R_{1}-R_{2}\right)}{\left(R_{C}+R_{1}\right)\left(R_{C}+R_{2}\right)}\left(\varepsilon_{Ni}-\varepsilon_{C}\right)$$

In order to verify the theoretical formula (16), one has to measure the resistance of the yarns involved in our experiment. The results are presented in Table 2 [13].

From the SEM image, it can be considered that Ni was completely removed in the etching solution containing 10% H_2_O_2_ + 37% HCl (1:1). The electric resistance is then just the resistance of the carbon or:

(17) $$R_{C}=45.933\;\Omega /m$$

The non-etched part has a resistance of 2.267 Ω/m, which is the parallel connection of $R_{C}$ and $R_{2}$, or:

(18) $$\frac{1}{R_{C}}+\frac{1}{R_{2}}=\frac{1}{2.2667}\;\mathrm{or}\;R_{2}=2.3844\;\Omega /m$$

Similarly, one can calculate the value of $R_{1}$ for 6% H_2_O_2_ and 37% HCl (1:1) etching:

(19) $$\frac{1}{R_{C}}+\frac{1}{R_{1}}=\frac{1}{31.533}\;\mathrm{or}\;R_{1}\left(6\%\right)=100.584\;\Omega /m$$

and for 3% H_2_O_2_ and 37% HCl (1:1) etching:

(20) $$\frac{1}{R_{C}}+\frac{1}{R_{1}}=\frac{1}{2.867}\;\mathrm{or}\;R_{1}\left(3\%\right)=3.0575\;\Omega /m$$

**Figure 5:**
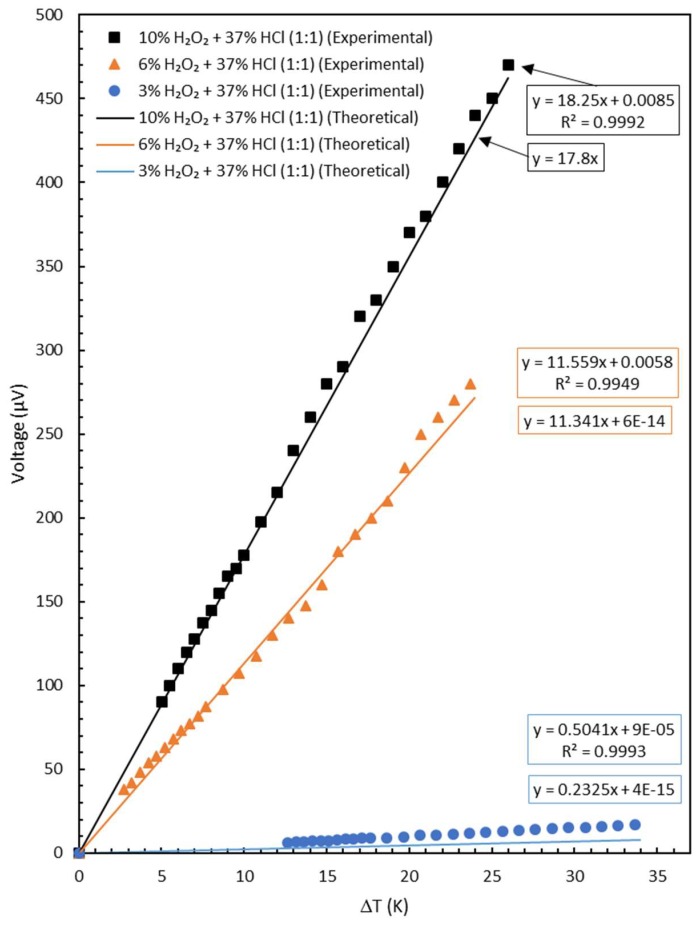
Plot of voltage vs. temperature difference of the samples (theoretical and experimental).

Inserting all the known values into the Equations (12) and (16), one can plot the theoretical graphs as shown in Figure 5 (with lines). The graph for the sample treated with 10% H_2_O_2_ + 37% HCl was calculated with Equation (12) because *S*_1_ was considered equal to 0. The graphs for the 3% H_2_O_2_ + 37% HCl and 6% H_2_O_2_ + 37% HCl were calculated with Equation (16). In Figure 5, the graph of the experimental data (with markers) was also presented as a comparison to the theoretical one.

Values inside the boxes are linear trend line equations for the corresponding data of the 3–10% H_2_O_2_ with which each was mixed with 37% HCl in 1:1 ratio. The orange and blue boxes are attributed to the experimental and theoretical trend lines, respectively. The Seebeck coefficient values were taken from the slope of voltage vs. temperature difference.

It is clear from Figure 5 that the agreement between the theoretical and experimental data is quite good for the yarns etched with 6% and 10% H_2_O_2_. The case with 3% H_2_O_2_ shows a poor agreement. It must be pointed out, however, that 3% H_2_O_2_ was also a very poor etching. This is proved by the resistance values presented in Table 2. The linear electrical resistance only changed from 2.2667 to 2.8667 Ω/m or 26%.

The Equation (16) can be rearranged as:

(21) $$V_{0}=\left(T_{H}-T_{C}\right)\left(\varepsilon_{Ni}-\varepsilon_{C}\right)\frac{\frac{R_{C}}{R_{2}}\left(\frac{R_{1}}{R_{2}}-1\right)}{\left(\frac{R_{C}}{R_{2}}+\frac{R_{1}}{R_{2}}\right)\left(\frac{R_{C}}{R_{2}}+1\right)}$$

**Figure 6:**
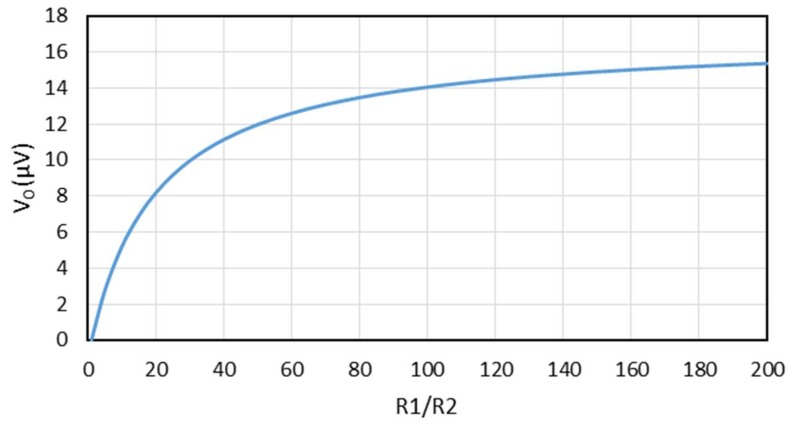
The generated voltage $V_{0}$ versus $R_{1}/R_{2}$ plotted from Equation (21) with a temperature difference of 1 K.

If we insert the values from Equations (17)–(20) into the theoretical formula (21), we can make a plot of the generated voltage $V_{0}$ versus $R_{1}/R_{2}\;$ as shown in Figure 6. One observes that the value of $R_{1}$ can have a dramatic influence on the overall performance of this thermocouple.

## 4. Conclusions

In this work, a combination of etched and non-etched nickel-coated carbon yarn (NiCCY) was used as the conductive materials for creating textile-based thermocouple. After performing the stripping process in three different concentration of stripping solutions, it is obvious that the higher the concentration of H_2_O_2_, the more intense the etching process was, with less Ni remaining on the surface of CF. From the theoretical calculation and experimental data, it is proved that there is a good agreement between the theoretical and experimental data, especially for the yarns etched with 6% and 10% H_2_O_2_ (both are mixed with 37% HCl). The yarn etched with 3% H_2_O_2_ shows a poor agreement due to the very poor etching action of the chemicals at 3% H_2_O_2_ and 37% HCl. The 10% H_2_O_2_ + 37% HCl was efficient enough to etch the Ni from NiCCY. We can conclude that the more efficient the etching process, the better the Seebeck coefficient created from the etched and non-etched NiCCY is. The R-squares of the experimental graphs are all more than 0.99, showing that the data are very close to the fitted regression line. The value of $R_{1}$ has a great influence on the whole characteristics of this thermocouple. Overall, we are successful in developing a theoretical formula to calculate the Seebeck coefficient of thermocouples made of etched and non-etched NiCCY based on the resistance value of the samples due to the simplicity of electrical resistance measurement.

## Figures and Tables

**Table 1:** Characteristics of nickel-coated carbon yarn (NiCCY) [14]*.*

Parameter	Description
Raw material	Carbon
Liner density [tex]	1420 tex
Coating	0.25 µm of Ni
No. of filaments	1200
Filament diameter [µm]	7.5 incl. Coating
Density [g/cm^3^]	2.70
Twist [tpm, type]	0
Linear electrical resistance [Ω/m]	2.2667
Commercial name	Tenax^®^-J HTS40

**Table 2:** Linear electrical resistance of etched and non-etched yarns [13]*.*

Etching Condition	Linear Electrical Resistance [Ω/m]
Non-etched	2.2667
3% H_2_O_2_ + 37% HCl (1:1)	2.8667
6% H_2_O_2_ + 37% HCl (1:1)	31.533
10% H_2_O_2_ + 37% HCl (1:1)	45.933
